# The evolutionary history of *Plasmodium falciparum* from mitochondrial and apicoplast genomes of China-Myanmar border isolates

**DOI:** 10.1186/s13071-024-06629-3

**Published:** 2024-12-30

**Authors:** Yini Tian, Run Ye, Dongmei Zhang, Yilong Zhang

**Affiliations:** Department of Tropical Diseases, Faculty of Naval Medicine, Naval Medical University, Shanghai, 200433 China

**Keywords:** Evolutionary history, China-Myanmar border, Mitochondrial, Apicoplast, *Plasmodium falciparum*

## Abstract

**Background:**

The frequent communication between African and Southeast Asian (SEA) countries has led to the risk of imported malaria cases in the China-Myanmar border (CMB) region. Therefore, tracing the origins of new malaria infections is important in the maintenance of malaria-free zones in this border region. A new genotyping tool based on a robust mitochondrial (*mt*) /apicoplast (*apico*) barcode was developed to estimate genetic diversity and infer the evolutionary history of *Plasmodium falciparum* across the major distribution ranges. However, the *mt*/*apico* genomes of *P. falciparum* isolates from the CMB region to date are poorly characterized, even though this region is highly endemic to *P. falciparum* malaria.

**Methods:**

We have sequenced the whole *mt/apico* genome of 34 CMB field isolates and utilized a published data set of 147 *mt/apico* genome sequences to present global genetic diversity and to revisit the evolutionary history of the CMB *P. falciparum*.

**Results:**

Genetic differentiation based on *mt/apico* genome of *P. falciparum* revealed that the CMB (Lazan, Myanmar) isolates presented high genetic diversity with several characteristics of ancestral populations and shared many of the genetic features with West Thailand (Mae Sot; WTH) and to some extent West African (Banjul, Gambia; Navrongo, Ghana; WAF) isolates. The reconstructed haplotype network displayed that the CMB and WTH *P. falciparum* isolates have the highest representation (five) in the five ancestral (central) haplotypes (H1, H2, H4, H7, and H8), which are comparatively older than isolates from other SEA populations as well as the WAF populations. In addition, the highest estimate of the time to the Most Recent Common Ancestor (TMRCA) of 42,400 (95% CI 18,300–82100) years ago was presented by the CMB *P. falciparum* compared to the other regional populations. The statistically significant negative values of Fu's *F*s with unimodal distribution in pairwise mismatch distribution curves indicate past demographic expansions in CMB *P. falciparum* with slow population expansion between approximately 12,500–20,000 ybp.

**Conclusions:**

The results on the complete *mt/apico* genome sequence analysis of the CMB *P. falciparum* indicated high genetic diversity with ancient population expansion and TMRCA, and it seems probable that *P. falciparum* might have existed in CMB, WTH, and WAF for a long time before being introduced into other Southeast Asian countries or regions. To reduce the impact of sample size or geographic bias on the estimate of the evolutionary timeline, future studies need to expand the range of sample collection and ensure the representativeness of samples across geographic distributions. Additionally, by mapping global patterns of *mt/apico* genome polymorphism, we will gain valuable insights into the evolutionary history of *P. falciparum* and optimised strategies for controlling *P. falciparum* malaria at international borders.

**Graphical Abstract:**

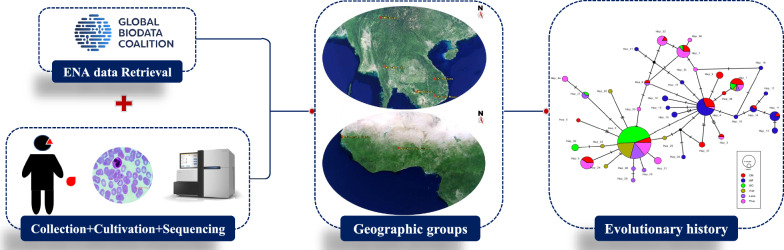

**Supplementary Information:**

The online version contains supplementary material available at 10.1186/s13071-024-06629-3.

## Background

*Plasmodium falciparum* ranks as the most lethal parasite globally, accounting for most malaria-associated deaths [1]. Despite a significant reduction in the global malaria burden attributed to *P. falciparum* following the adoption of artemisinin-based combination therapies (ACTs) as the primary treatment in all endemic areas, the development of resistance to these drugs remains a significant impediment to controlling the disease [2, 3]. Moreover, the frequent human migration across countries where malaria is endemic promotes the transmission of increasingly virulent and resistant strains of malaria in novel ecological and epidemiological niches [4].

Various population genetic studies have corroborated these events by performing comprehensive sequencing of both nuclear and mitochondrial (*mt*) genomes or by identifying single-nucleotide polymorphisms (SNPs) in putatively neutral DNA regions within the nuclear genomes of *P. falciparum* or *Plasmodium vivax* [5–7]. Distinct from the nuclear genome, the apicoplast (*apico*) and *mt* serve as vital intracellular organelles within apicomplexan parasites [8]. The stringent maternal inheritance of these extranuclear genomes precludes recombination, thus offering a stable and geographically informative genetic marker or barcode over time [9]. Earlier studies have demonstrated the utility of *mt* genes for phylogenetic analysis [6, 10–12]. In a similar vein, the *apico* has been employed to investigate and reconstruct evolutionary histories. Crucially, the *apico*, emerging from secondary endosymbiosis among ancestral apicomplexan parasites, harbors unique metabolic pathways critical for the survival of the parasite and distinct from those of the host [13, 14]. Owing to its single copy and enhanced phylogenetic information relative to the *mt* genome, genes in the *apico* genome have been frequently utilized as markers in *Plasmodium* phylogeny studies [15]. Consequently, in view of the non-recombining and co-inherited properties of the *apico* and *mt* genomes, researchers have developed robust barcoding methodologies to monitor the global dispersal of *Plasmodium* species, combining SNPs from both genomes [9, 16].

Although malaria was eradicated in China by 2021, ongoing communication with African and the Greater Mekong Subregion (GMS) countries, along with a rise in cross-border travel, persistently exposes the country to the risk of imported malaria cases [17]. The frequency of imported malaria cases has been consistently increasing because of the growing international exchange activities, especially along border regions, such as the China-Myanmar border (CMB) [18, 19]. In regions declared malaria-free, it is essential to identify the origins of new infections to preserve these zones and ensure immediate reactions to individual malaria cases [20]. Therefore, in this study, we conducted population genetic analysis based on the 34 *mt/apico* genomes of *P. falciparum* isolates from the CMB alongside 147 *mt/apico* genomes from five different global sites: West Africa (WAF), West Thailand (WTH), West Cambodia (WKH), Vietnam (VN), and Laos (LA). The aim of this study is to investigate the genetic interrelationships among different *P. falciparum* isolates and deepen our understanding on the evolutionary history of global *P. falciparum*. By conducting detailed *mt/apico* genome sequencing of *P. falciparum* isolates from the CMB, we anticipate gaining new insights into the global evolutionary patterns of this malaria pathogen.

## Methods

### Data acquisition and summary statistics

We previously reported the whole genome sequencing and genetic diversity based on 14 chromosomes of 34 *P. falciparum* isolates from Nabang-Lazan valley (longitude: 97°33′47.81″; latitude: 24°45′21.65″) on the China-Myanmar border (CMB) [20]. Samples exhibited mixed infections according to estMOI software (MOI < 1 or MOI = 1) and moimix package of R (FWS > 0.95), and low coverages < 30-fold or < 70% were finally excluded from the analysis [21, 22]. Details of the sequence's accession of included samples are given in Additional file 1 (Table S1). The *mt/apico* genome sequences were obtained from the genome sequencing data of the included samples for further analysis. Potential SNPs were identified using GATK according to certain criteria [20].

Pairwise *F*_ST_ calculations for estimating population differentiation based on haplotype frequency differences, along with Nei’s Nm estimated gene flow conformed to GST [23], were conducted using Arlequin v.3.5. In addition, the distribution of genetic variation in population was also calculated through 1000 permutations in Arlequin v.3.5 by using an analysis of molecular variance (AMOVA) [24]. Furthermore, by using a spatial analysis of molecular variance (SAMOVA 2.0), these *mt/apico* genome sequences were clustered into geographically and genetically homogeneous groups or populations [25]. SAMOVA generates F-statistics (*F*_CT_, *F*_ST_, *F*_SC_), using the AMOVA approach, to K group to maximize group variations. The SAMOVA estimate was calculated for K = 2–6, with 1000 simulated annealing steps from each of 100 sets of initial starting conditions. An evaluation of isolation by distance (IBD) was conducted using a nonparametric Mantel test with the assistance of the web-based IBDWS v.3.16 program [26].

### Reconstruction of mt genome haplotype networks

To investigate the demographic patterns of *P. falciparum* at the CMB, we performed a mismatch distribution analysis with DnaSP v5. This method involves comparing the observed mismatch distributions to those predicted under a model of sudden demographic expansion, using parametric bootstrap analysis with 1000 replicates. The smoothness of the mismatch distribution was quantified by calculating the raggedness index (r) [27].

Using MEGA X [28], a maximum likelihood (ML) phylogenetic tree was constructed with 1000 bootstraps. The number of segregating sites (S), number of haplotypes (h), haplotype diversity (Hd), and nucleotide diversity (Pi) were computed using DnaSP v5 [29–31]. The *mt/apico* genome sequences of *P. falciparum* from different global isolates were tested for neutrality and recombination using Tajima’s *D* [32], Fu and Li’s *D*, and Fu and Li’s *F* [33] statistics in DnaSP v5 to determine their suitability for evolutionary studies. Statistical significance was determined through 1000 coalescent simulations under a standard neutral model of molecular evolution without recombination [31].

### Inference of phylogeography

The Bayesian Markov chain Monte Carlo (MCMC) approach in BEAST v1.10.4 was used to estimate the present effective population size and TMRCA for both the global and CMB populations of *P. falciparum* [34]. The 'NEX' file obtained by concatenating *mt* and *apico* gene using the 'concatenate sequence' program function in PhyloSuite was imported into BEAST v1.10. An MCMC Bayesian analysis was performed using BEAST v1.10.4, incorporating the Hasegawa-Kishino-Yano (HKY) model, an uncorrelated relaxed clock model, and a Bayesian Skyline prior. The analysis was run with a chain length of 50,000,000 and with states recorded every 10,000 iterations. To enhance the reliability and robustness of TMRCA, we replaced the HKY model with the General Time Reversible (GTR) model and recalculated the TMRCA. First, 10% of iterations were discarded as burn-in. Log files were assessed using TRACER 1.7.2 (http://tree.bio.ed.ac.uk/software/tracer/) to ensure that all statistics had attained effective sample sizes of > 200. If ESS < 200, optimization was employed by adding 500,000 iterations (chain length) each time. The results were visualized using TRACER v1.7.2, with parameter uncertainties expressed via 95% confidence intervals (CI) and/or 95% highest probability density (HPD).

## Results

### Genetic variation and recombination across the whole *mt/apico *genomes of *P. falciparum*

We previously reported the whole genome sequencing of 34 *P. falciparum* isolates from Nabang-Lazan valley at the China-Myanmar border (CMB) [20]. After preprocessing the data as outlined previously, the *mt/apico* genome sequences from the 34 isolates were further analyzed to identify potential SNPs using GATK [20]. To conduct a broader comparison of haplotypes among diverse geographic regions and to clarify the global evolutionary trajectory of *P. falciparum*, we examined data from the ENA database from neighboring SEA countries as well as West Africa countries (Additional file 1: Table S1). In total, 181 *mt/apico* genomes including 34 sequences in this study and 147 sequences from the ENA database were analyzed for six populations: CMB (*n* = 34), WAF (*n* = 33), WKH (*n* = 38), WTH (*n* = 40), VN (*n* = 19), and LA (*n* = 17) (Table 1). A series of quality control measurements was employed to discard SNPs susceptible to high-risk genotypic errors (see Materials and Methods). After these procedures, 1,43 high-quality SNPs were identified, 41 of which exhibited a minor allele frequency (MAF) exceeding 0.01 (data not shown). Based on the 41 SNPs, 38 haplotypes across six geographical populations were constructed, yielding Hd and Pi values of 0.832 and 0.06097, respectively (Table 1). Notably, CMB isolates demonstrated higher Hd (0.930) compared to other populations: 0.826 (WAF), 0.333 (WKH), 0.468 (VN), 0.662 (LA), and 0.877 WTH (Table 1). Nucleotide diversity showed a similar pattern, with Pi values of 0.06956 (CMB), 0.05959 (WAF), 0.01978 (WKH), 0.02216 (VN), 0.03336 (LA), and 0.06954 (WTH) (Table 1).

**Table 1:** Genetic diversity indices and neutrality tests (Fu’s *Fs* and Tajima’s *D*) based on mt/apico genome of *P. falciparum*

Groups	n	Haplotype code	S	Pi	h	Hd	k	Fu's *Fs*	Tajima’s *D*
Total	181	H1(11), H2(69), H3(2), H4(20), H5(1), H6(2), H7(11), H8(10), H9(3), H10(2), H11(6), H12(2), H13(2), H14(3), H15(1), H16(1), H17(1), H18(1), H19(1), H20(1), H21(3), H22(1),H23(1), H24(1), H25(1), H26(1), H27(3), H28(1), H29(1), H30(3), H31(3), H32(6), H33(1), H24(2), H35(1), H36(1), H37(2), H38(1)	38	0.06097	38	0.832	2.317	−31.726^***^	−1.89814
CM	34	H1(5), H2(4), H3(1), H4(6), H5(1), H6(1), H7(3), H8(4), H9(3), H11(1), H14(1), H32(1), H37(2), H38(1)	20	0.06956	15	0.930	2.852	−6.309^**^	−1.41902
WAF	33	H4(13), H10(2), H11(5), H12(2), H13(2), H14(2), H15(1), H16(1), H17(1), H18(1), H19(1), H20(1), H21(1)	16	0.05959	13	0.826	2.433	−5.064^**^	−1.26746
WKH	38	H1(2), H2(31), H7(1), H27(1), H30(3)	8	0.01978	5	0.333	0.811	−0.940	−1.65525
VN	19	H1(1), H2(14), H22(1), H23(1), H24(1), H25(1)	8	0.02216	6	0.468	0.842	−2.894^*^	−2.16199^**^
LA	17	H1(1), H2(10), H6(1), H26(1), H27(2), H28(1), H29(1)	9	0.03336	7	0.662	1.368	−2.801^*^	−1.75275
WTH	40	H1(2), H2(10), H3(1), H4(1), H7(7), H8(6), H31(3), H32(5), H33(1), H34(2), H35(1), H36(1)	16	0.06954	12	0.877	−2.502	−2.502^*^	−0.77834

n.d., not determined; #*p* < 0.10; *p < 0.05; **p < 0.02; ***p < 0.001

*n*, number of sequences, *S* number of polymorphic sites, *Pi* nucleotide diversity, *h* number of haplotypes, *Hd* haplotype diversity, *CMB* China-Myanmar border, *WAF* West Africa, *LA* Laos, *VN* Vietnam, *WTH* West Thailand, *WKH* West Cambodia

To reduce biases in assessing genetic diversity due to recombination or natural selection, we investigated these phenomena within the *mt/apico* genomes of CMB and other geographic *P. falciparum* isolates using DnaSP v5 [31]. We found no recombination events (Rm = 0) [35]. Moreover, an analysis of linkage disequilibrium (LD) and physical distance (r^2^ = 0.0664, *p* > 0.05) indicated that adjacent nucleotide sites are no more correlated than distant sites.

### Population structure and genetic differentiation based on *mt/apico *genome of *P. falciparum*

**Fig. 1:**
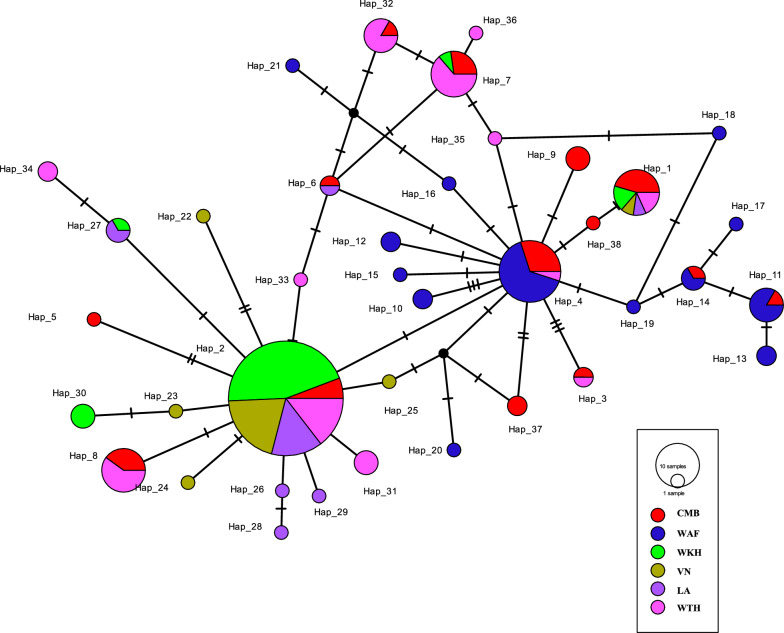
Reconstructed haplotype network of global *Plasmodium falciparum* isolates with whole *mt/apico* genome sequences. China-Myanmar border haplotypes are indicated by red color, West Africa by blue, West Cambodia by green, Vietnam by yellow, Laos by purple, and West Thailand by pink. The size of the circle is proportional to the haplotype frequency

The median-joining network, representing 181 *mt/apico* genomes, highlighted the distribution of 38 haplotypes across the *P. falciparum* population (Fig. 1). Five central (ancestral) haplotypes—H1, H2, H4, H7, and H8—exhibited high outgroup weights (*p* > 0.05), shared predominantly among multiple regions: H1 by CM/WTH/WKH/VN/LA, H2 by WKH/VN/WTH/LA/CM, H4 by WAF/CM/WTH, H7 by WTH/CM/WKH, and H8 by WTH/CM. *Plasmodium falciparum* populations formed three main clusters: Cluster 1, centered around H2, excluded WAF; Cluster 2, centered around H4, included primarily WAF and CMB; Cluster 3, centered around H7, was dominated by WTH and CMB. Within Cluster 1, the WKH population harbored a significant proportion (44.92%) of H2, followed by VN (20.30%), LA (14.49%), WTH (14.49%), and CMB (5.80%). Conversely, WAF predominantly harbored H4 (65.00%), with lesser contributions from CMB (30.00%) and WTH (5.00%). In Cluster 3, WTH held a majority (63.63%) of H7, followed by CMB (27.27%) and WKH (9.10%). Those characteristics of phylogeny in the median-joining network were also reflected in the ML phylogenetic tree analysis (Additional file 2: Figure S1).

AMOVA analysis indicated that most genetic variance occurred within populations (75.53%) rather than among groups (21.99%) or among populations within groups (2.49%), pointing to pronounced geographic clustering. Significant differences were observed in the fixation index among groups (*F*_CT_), among populations within groups (*F*_SC_), and within populations (*F*_ST_) (Additional file 3: Table S2).

Distinct genetic differentiation was found between CMB and WKH populations (*F*_ST_ = 0.22023, *p* < 0.05), CMB and VN populations (*F*_ST_ = 0.19374, *p* < 0.05), and CMB and LA populations (*F*_ST_ = 0.14779, *p* < 0.05). In contrast, lesser differentiation was identified between CMB and WAFGH populations (*F*_ST_ = 0.06915, *p* < 0.05) and between CMB and WTH populations (*F*_ST_ = 0.05953, *p* < 0.05). High levels of gene flow were detected between CMB and WAFGH populations (Nm = 6.73109) and between CMB and WTH populations (Nm = 7.89971), but these were not evident between CMB and other SEA populations (Additional file 4: Table S3).

### Spatial genetic structure analysis, demographic history, and neutrality test

The spatial genetic structure of *P. falciparum* based on *mt/apico* genomes was assessed by SAMOVA analysis, which assigned cluster numbers (K values) from 2 to 6. Within this range, a decrease in the *F*_CT_ value, indicating genetic differences among groups, was observed from K = 2 to K = 3. Conversely, an increase in the *F*_CT_ value occurred from K = 3 to K = 6, while *F*_*SC*_ and *F*_ST_ values, reflecting genetic differences within and among populations, consistently decreased. Therefore, K = 3 was deemed the most suitable clustering parameter. Under this classification, Group 1 included CMB and WTH populations; Group 2 comprised WAFGM and WAFGH populations; Group 3 covered other SEA populations, specifically WKH, VN, and LA (Additional file 5: Table S4) (Additional file 6: Figure S2). These divisions were corroborated by results from the median-joining network and phylogenetic analyses (Fig. 1, Additional file 2: Figure S1). A Mantel test established a robust correlation between geographic and genetic distances among all populations (Z = 39,272.3373, r = 0.7524, one-sided *p* < = 0.0040), suggesting the influence of geographical separation on genetic structuring (Additional file 7: Figure S3a). Significant correlations were also detected specifically between CMB and WAF populations as well as between CMB and SEA populations (Additional file 7: Figure S3b).

**Fig. 2:**
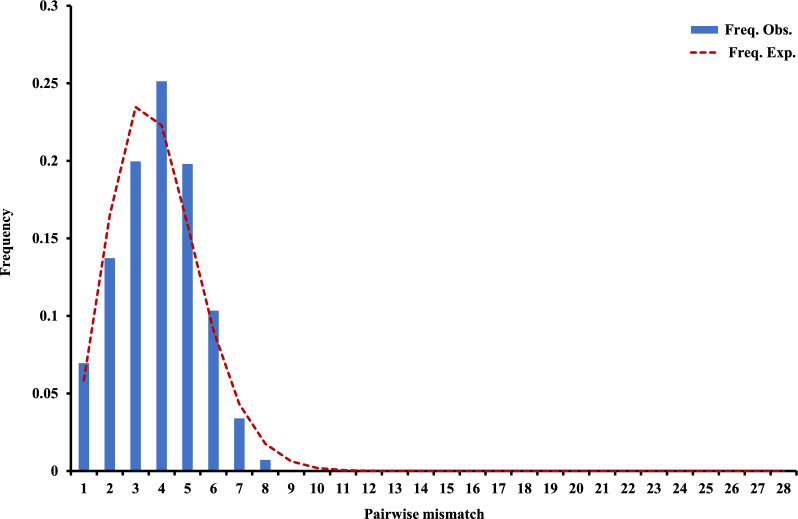
Mismatch distribution of pairwise number of differences at the China-Myanmar border for *Plasmodium falciparum*. The bars represent observed frequency of the pairwise differences among *mt/apic*o genome sequences, and the line represents the expected curve for a population that has undergone a demographic expansion. Observed mismatch distribution was compared with that expected under the sudden demographic expansion model with a parametric bootstrap of 1000 replicates

Neutrality tests (Fu’s *Fs* and Tajima’s *D*) on the 181 *mt/apico* genomes of *P. falciparum* isolates revealed that the CMB population exhibited significant negative neutrality test results (Tajima’s *D* = −1.41902, *p* > 0.05; Fu’s *Fs* = -6.309, *P* < 0.02) (Table 1), indicating a demographic expansion subsequent to a bottleneck event, as evidenced by a smooth and unimodal mismatch distribution (Fig. 2). Deviations from neutrality were significant across all other geographic populations except WKH, as determined by either Fu’s *Fs* or Tajima’s *D* tests (Table 1). Given Fu’s *Fs*'s sensitivity to demographic shifts, discerning whether the observed patterns are a result of positive selection or demographic events like population expansion remains challenging.

### Inference of phylogeography of CMB *P. falciparum*

**Fig. 3:**
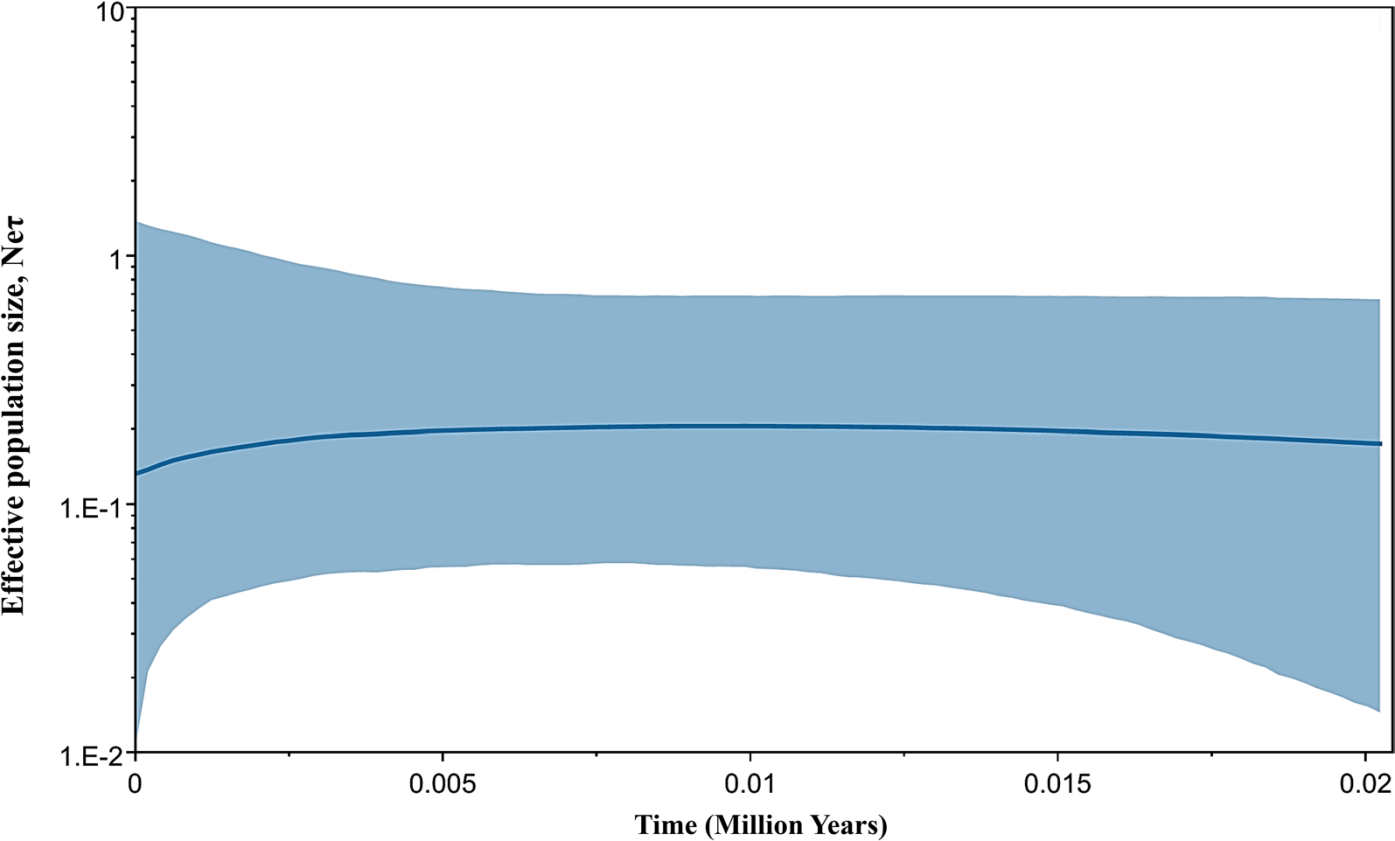
Bayesian skyline plot of China-Myanmar border *Plasmodium falciparum* showing changes in effective population size (Ne) through time. The Y-axis represents the effective population size on a logarithmic scale, and the X-axis represents time thousands of years ago. The thick solid blue line is the median estimate, and the blue shaded area represents the 95% highest probability density (HPD) intervals for effective population size

Exploring the past population dynamics of *P. falciparum* isolates at the CMB, specifically examining temporal changes in effective population size, involved using Bayesian skyline plot analysis. It is estimated that the effective population size of *P. falciparum* along this border region began to expand approximately 12,500–20,000 years before present (ybp) (Fig. 3). The TMRCA of the CMB *P. falciparum* population (42, 400 ybp, 95% CI; 8300–82100 ybp) exceeds those from WAF, WTH, VN, LA, and WKH (Table 2) under the HKY model. To enhance the reliability and robustness of TMRCA, we recalculated the TMRCA by using the GTR model. The calculation results generated from GTR are similar to those from the HKY model (Table 2).

**Table 2:** TMRCA and the current effective population size of regional and global *Plasmodium falciparum* isolates as estimated by the BEAST computer program

Region	TMRCA in Mya (L-U 95% CI)	
	HKY model	GTR model
China-Myanmar border	0.0424 (0.0183–0.0821)	0.0444 (0.0192–0.0857)
West Africa	0.0375 (0.0164–0.0768)	0.0394 (0.0165–0.0787)
Laos	0.0387 (0.0174–0.0726)	0.0403 (0.0182–0.0755)
Vietnam	0.0374 (0.0172–0.0705)	0.0387 (0.018–0.0731)
West Thailand	0.0406 (0.0184–0.0779)	0.0425 (0.0193–0.0815)
West Cambodia	0.0400 (0.0185–0.0768)	0.0415 (0.0192–0.0788)
All with CM isolates	0.0430 (0.0184–0.0841)	0.0449 (0.0195–0.0872)
All without CM isolates	0.0429 (0.0184–0.0838)	0.0445 (0.0193–0.0901)

*L* lower value, *U* upper value, 95% CI, 95% confidence interval

HKY, Hasegawa-Kishino-Yano model

GTR, General Time Reversible model

## Discussion

The international dispersal of *P. falciparum* parasites significantly jeopardizes elimination initiatives in malaria-endemic regions and the effectiveness of malaria treatments [1]. However, the lack of geographical specificity and frequent recombination, disrupting multi-locus SNP associations in each generation, renders nuclear SNP barcoding inadequate for reconstructing this parasite's evolutionary history [36]. On this basis, various investigations have applied *mt* DNA to uncover new *Plasmodium* species, clarify phylogenetic relationships, and study historical and biogeographical patterns [6, 11, 37–50]. The *mt* haplotype distribution points to an African genesis of *P. falciparum*, followed by its dispersion to SEA and South America (SAM) [11], likely mirroring proposed human migration routes [11, 41]. This theory is reinforced by the high genetic diversity found in African *P. falciparum* populations compared to others worldwide [6]. Yet, *mt* haplotypes by themselves are inadequate for accurately determining the geographic origins of parasite isolates without a loss of specificity in geographical assignment [9].

Prior research revealed that *apico* sequences display greater polymorphism than *mt* sequences, attributed to their larger size. Given the analogous A + T content between the *apico* and *mt* genomes across Apicomplexa species and their uniparental inheritance [42], multiple studies have combined *mt* and *apico* loci data to construct a more robust phylogeny for certain *Plasmodium* parasites [9, 16, 43]. Accordingly, a novel genotyping approach using a robust *mt/apico* barcode has been developed, withstanding recombination’s diluting effects [9]. The integration of *apico* SNPs to establish *mt/apico* haplotypes markedly improves the geographic resolution of the samples, for example, six of the eight widely observed *mt/apico* haplotypes are most frequently detected in Africa (WAF and East Africa), confirming the African origin of *P. falciparum* [9]. Additionally, thorough analysis of *apico* SNP variations across different *P. falciparum* populations will enhance our comprehension of *apico* evolution and aid in determining global diversity and recombination patterns [9]. Thus, by mapping global patterns in organellar genome polymorphisms, we will gain valuable insights into how *P. falciparum* populations worldwide are interconnected by international malaria migration [9]. Unfortunately, similar research has yet to be undertaken in the CMB region, a key area within the GMS. For this study, to assess genetic diversity and other population genetic parameters, we analyzed the complete *mt* and *apico* genomes of 34 *P. falciparum* isolates collected from the CMB as well as the sequence data of 147 *P. falciparum* isolates from various endemic regions by retrieving ENA database. This expanded our analysis of the worldwide evolutionary history of this highly virulent malaria parasite. Our findings first revealed that the CMB *P. falciparum* displays greater genetic diversity in the *mt/apico* genome than seen in other global isolates (Table 1). However, it should be noted that the use of a relatively low genome coverage threshold (compared to 90%) may lead to insufficient coverage and inaccurate conclusions about SNP presence or absence, thereby affecting the assessment of genotype accuracy. Thus, additional caution is required when processing and analyzing the NGS data. In this case, after evaluating the quality of our previous NGS data, the threshold of NGS coverage was set to > 70%, which is considered relatively reasonable and to some extent explains the results, in both the 2019 study [20] and the present study.

However, various evolutionary genetic studies used mitochondrial genome sequences to conduct molecular dating analyses by assuming strict molecular clock models with fixed calibrations [44–47]. However, time estimates on the mitochondrial DNA are not robust to model and parameter perturbations. They are found to be affected by the fundamental assumptions of the dating methods [46–48]. Using simple timing methodologies with strong assumptions might lead to biases in the estimated times, even when good calibration points are available [49]. Therefore, the application of more complex assumptions and methodologies which compensate for variable evolutionary rates such as relaxed clock methods would be needed to estimate a timeline for the evolution of parasites. Furthermore, the uncertainty of time estimates could be reduced when including nuclear and/or apicoplast genes to the analyses [49]. Liu’s single-genome amplification (SGA) study provides a different perspective on the evolutionary timeline of *P. falciparum* compared to clocks strictly tied to species differentiation events, suggesting a more recent origin for *P. falciparum* in humans [50]. Pacheco's work on *Plasmodium* in lemurs notably revealed that the TMRCA calculations, derived from the combined *mt* and *apico* genomes, yielded similar yet modestly younger time estimates than those from mtDNA alone, exhibiting distinct outcomes within the Laverania subgenus clade and featuring more constrained credibility intervals compared to mtDNA [43]. Nevertheless, TMRCA analyses employing both *mt* and *apico* genomes in *P. falciparum* are scarce. In the current analysis, by employing *mt/apico* genomic data, the highest TMRCA estimation of 42 400 years ago (95% CI; 18,300–82100) was recorded for the CMB *P. falciparum*, compared to the other regional populations (Table 1) and antecedes human settlement in Southeast Asia [51]. Incorporating the CMB isolates into the overall TMRCA calculation for the global population escalated the estimate to 43,000 years ago (95% CI; 18,400–84,100). Interestingly, the results consistently showed a lower TMRCA for Africa compared to Asia in this study by using either strict or relaxed molecular clock models. However, these results are consistent with previous studies, suggesting that the Africa strain might have experienced a rapid population expansion in a relatively recent historical period [6, 11]. The variances in TMRCA values across these analyses and this study likely stem from diverse sample sizes and employing different organellar genomes. More important is the ‘effective population size’ in the calculation of TMRCA. Due to a possible local epidemic expansion, even parasites collected from different patients and/or different villages might exhibit the same or similar genetic backgrounds. False calculation of TMRCA will be processed if including these parasites in the analyses [52]. To confirm the accuracy and reduce the impact of sample size or geographic bias on the results, it may be expedient to expand the range of sample collection and ensure the representativeness of samples across geographic distributions in future studies [52]. Besides, the analysis of TMRCA covering all major geographic regions will ensure consistency in statistical methods, software tools, and parameters used, thus minimizing differences in TMRCA estimates that may arise from variations in methods and parameters. Despite these discrepancies, the oldest estimated TMRCA pointing toward ancestral natures of *P. falciparum* from the CMB remains unassailable in this study.

While exploring the overall genetic diversity and phylogeographic patterns, examining the historical demography of populations reveals insights into their evolutionary histories [7, 53]. Past demographic expansions of the CMB *P. falciparum* populations are shown by the unimodal distribution in the pairwise mismatch distribution curves (Fig. 2) and the statistically significant negative values of Fu's *Fs* (Table 1). These expansions likely occurred between approximately 12,500 and 20,000 ybp (Fig. 3). Intriguingly, the timing of these expansions along the CMB closely aligns with those in Africa. While expansions in African *P. falciparum* populations may have commenced around 10,000 ybp [11], those at the CMB are estimated to have occurred slightly earlier. This expansion is hypothesized to have been facilitated by the advent of agricultural practices and the speciation of the malaria vector *Anopheles gambiae* during this period [11]. Supporting evidence of simultaneous expansions in *P. falciparum* populations across both the CMB and Africa underpins this hypothesis.

Furthermore, it is suggested that populations close to the ancestral species distribution range maintain higher genetic diversity than those at the periphery [6, 53, 54]. Therefore, it appears probable that the CMB *P. falciparum* populations constitute a part of this species’ ancestral distribution range. The reconstructed haplotype network further substantiates the ancestral status of the CMB *P. falciparum* isolates (Fig. 1). In the present study, the CMB has the highest representation (five) in the five ancestral (central) haplotypes (H1, H2, H4, H7, and H8), suggesting that the CMB *P. falciparum* is comparatively older than isolates from other SEA populations as well as the WAF populations. Notably, the *P. falciparum* isolates from WTH also constitute a part of the species' ancestral distribution range. According to the SAMOVA analysis, three geographic groups were identified, with CMB and WTH populations forming Group 1 (Additional file 2: Figure S1). Although the Mantel test confirmed a significant correlation between geographical and genetic distances among populations (Additional file 7: Figure S3), the low level of genetic differentiation (*F*_ST_ = 0.05953, *p* < 0.05) as well as high gene flow (Nm = 7.89971) between the CMB and WTH populations (Additional file 4: Table S3) reflects ongoing and frequent parasite migration between these areas, even though the distance between CMB and WTH is nearly 800 km (Additional file 8: Figure S4).

## Conclusions

The investigation of the entire *mt/apico* genome sequence of the CMB *P. falciparum* uncovers noteworthy genetic diversity with ancient population expansion and TMRCA. Therefore, it seems that the CMB *P. falciparum* is included in the ancestral distribution range of *P. falciparum*. Notably, the CMB *P. falciparum* exhibits numerous genetic similarities to isolates from WAF and WTH, yet it distinctly differs from other SEA *P. falciparum* populations. Given the closer genetic resemblance of CMB *P. falciparum* to WAF/WTH than to other SEA isolates, it is plausible that *P. falciparum* has been present in the CMB, WTH, and WAF regions long before spreading to other SEA countries. Recent and ongoing human migrations between CMB, WTH, and Africa may have further contributed to the observed genetic identity among these *P. falciparum* populations. To reduce the impact of sample size or geographic bias on the estimate of evolutionary timeline, future studies need to expand the range of sample collection and ensure the representativeness of samples across geographic distributions. Additionally, studying the population genetic characteristic of SNPs in the organellar genome of *P. falciparum* populations worldwide can provide valuable insights into the evolutionary history of this species and contribute to better strategies for controlling *P. falciparum* malaria.

### Additional data sets

The raw high-quality reads of the genomic data of parasite isolates from the other five endemic regions—WKH, West Cambodia (Pursat); WTH, West Thailand (Mae Sot); WAF, West Africa (Navrongo, Ghana; Banjul, Gambia); LA, Laso (Attapeu); VN, Vietnam (Binh Phuoc)—were downloaded from ENA (http://www.ebi.ac.uk/ena/) (Additional file 1: Table S1). The criteria for the selection of the comparator sequences of the isolates from ENA include > 1.0 G of sequencing data per isolate and genome average coverage > 30-fold.

## Supplementary Information

Additional file 1: Table S1. Information on 181 *mt/apico* genomes of *Plasmodium* *falciparum* from six geographical populations, including Lazan (China-Myanmar border, CMB), Banjul (Gambia, WAF-GM)/Navrongo (Ghana, WAF-GH), Pursat (West Cambodia, WKH), Mae Sot (West Thailand, WTH), Binh Phuoc (Vietnam, VN), and Attapeu (Laos, LA).

Additional file 2: Figure S1. Maximum likelihood phylogenetic tree of *Plasmodium* *falciparum* based on *mt/apico* genomes. Bootstrap values (1000 replicates) of maximum likelihood analyses are shown above/below the main lineages. Lineage designation is indicated on the right. Bars represent 8.0 substitutions per site based on *mt/apico* genomes. Different colors indicated different population groups of *P. falciparum*. CMB, China-Myanmar border; WAF, West Africa; LA, Laos; VN, Vietnam; WTH, West Thailand; WKH, West Cambodia.

Additional file 3: Table S2. Analysis of molecular variance (AMOVA) of six *Plasmodium* *falciparum* populations based on *mt/apico* genomes. *F*CT, fixation index among groups, FSC, among populations within groups, FST, within populations.

Additional file 4: Table S3. Genetic differentiation and gene flow among the geographic groups based on *mt/apico* genomes. The pairwise *F*_ST_ values and Nm values based on the *mt/apico* genomes are shown below and above the diagonal, respectively. Characters in bold indicated that the significance was *p* < 0.05). inf, infinite. CMB, Lazan (China-Myanmar border); WAF-GM, Banjul, Gambia (West Africa); WAF-GH, Navrongo, Ghana (West Africa); WKH, Pursat (West Cambodia); VN, Binh Phuoc (Vietnam); LA, Attapeu (Laos); WTH, Mae Sot (West Thailand).

Additional file 5: Table S4. Population groups identified by spatial analysis of molecular variance (SAMOVA) algorithm. Significant values ^*^p < 0.05; ^**^p < 0.01; ^***^p < 0.001, ns: Not significant. CMB, Lazan (China-Myanmar); WKH, Pursat (West Cambodia); VN, Binh Phuoc (Vietnam); LA, Attapeu, (Laos); WTH, Mae Sot (West Thailand); WAF-GM, Banjul (Gambia); WAF-GH, Navrongo (Ghana).

Additional file 6: Figure S2. The Y-axis represents the different F-statistics (FCT, FST, FSC). The red, blue, and black lines represent FCT, FST, and FSC, respectively. The X-axis represents the number of groups (K). SAMOVA estimate was calculated for K = 2-6, with 1000 simulated annealing steps from each of 100 sets of initial starting conditions.

Additional file 7: Figure S3. Isolation by distance, the relationship between geographical and genetic distances based on *mt/apico* genomes in *Plasmodium falciparum* populations. Isolation by distance (IBD) was examined using a nonparametric Mantel with the web-based computer program IBDWS v.3.16. (a) IBD of all six populations based on *mt/apico* genomes; (b) IBD between CMB and WAF populations based on *mt/apico* genomes; (c) IBD between CMB and SEA populations based on *mt/apico* genomes.

Additional file 8: Figure S4. Map of the populations from different geographical regions. CMB-Lazan, China-Myanmar border; WTH-Mae Sot, West Thailand; LA-Attapeu, Laos; WKH-Pursat, West Cambodia; VN-Binh Phuoc,Vietnam; WAF-Banjul, Gambia; WAFGH-Navrongo, Ghana. The map was prepared using LocaSpace Viewer.

## Data Availability

Data supporting the conclusions of this article are included within the article and its additional files. Illumina sequencing reads from this study are available in the European Nucleotide Archive (PRJEB32255). The SNP data are available in the European Variation Archive (PRJEB34415).

## Abbreviations

Apico: Apicoplasts
Mt: Mitochondria
GMS: Greater Mekong Sub-region
CMB: China-Myanmar border
ACTs: Artemisinin-based combination therapies
SNPs: Single-nucleotide polymorphisms
AMOVA: Analysis of molecular variance
SAMOVA: Spatial analysis of molecular variance
IBD: Isolation by distance
ML: Maximum likelihood
NJ: Neighbor-joining
TMRCA: The time to the most recent common ancestor
MCMC: Markov Chain Monte Carlo
ENA: European nucleotide archive
WAF: West Africa
WKH: West Cambodia
WTH: West Thailand
VN: Vietnam
LA: Laos
Hd: Haplotype diversity
Pi: Nucleotide diversity
R: Raggedness
ESS: Effective sample size
HPD: Highest probability density
CI: Confidence interval
SEA: South East Asia
SGA: Single-genome amplification
HKY: Hasegawa-Kishino-Yano
GTR: General time reversible
