# Supplementary figures and images for: The evolutionary history of Plasmodium falciparum from mitochondrial and apicoplast genomes of China-Myanmar border isolates
Source: Parasit Vectors. 2024 Dec 30;17:548. doi: 10.1186/s13071-024-06629-3 (PMC11686842; doi:10.1186/s13071-024-06629-3)

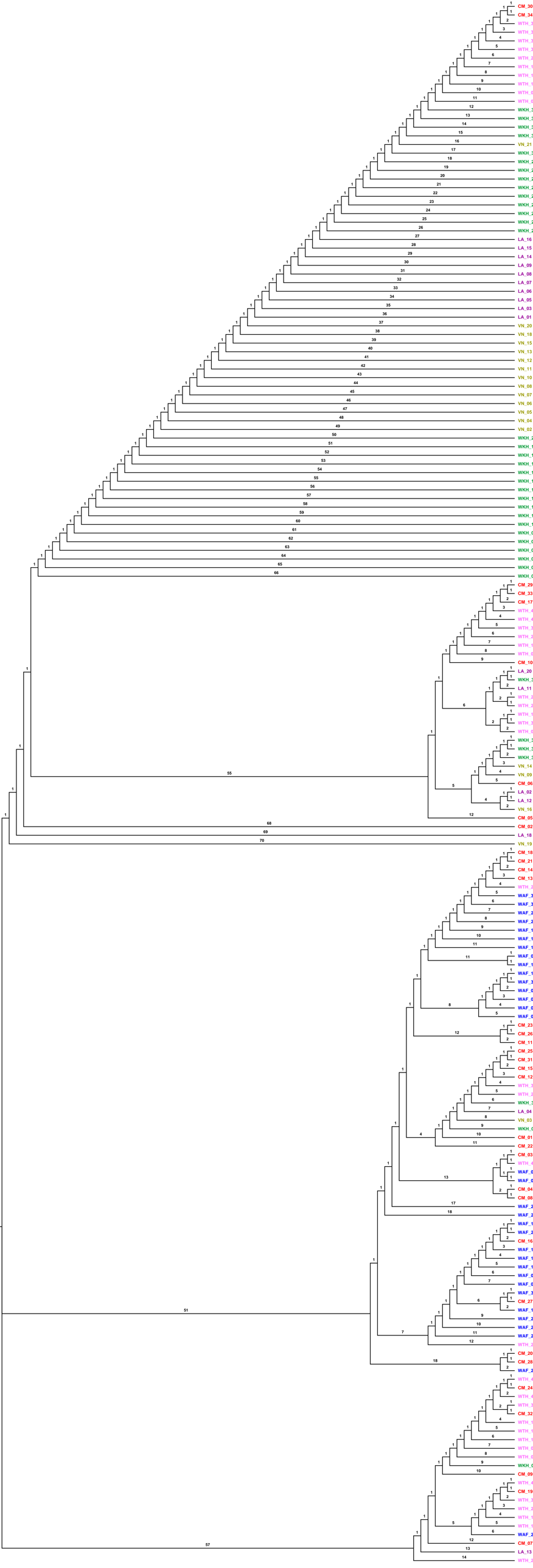

Supplement: Supplementary file 2 — Additional file 2: Figure S1. Maximum likelihood phylogenetic tree of Plasmodium falciparum based on mt/apico genomes. Bootstrap values (1000 replicates) of maximum likelihood analyses are shown above/below the main lineages. Lineage designation is indicated on the right. Bars represent 8.0 substitutions per site based on mt/apico genomes. Different colors indicated different population groups of P. falciparum. CMB, China-Myanmar border; WAF, West Africa; LA, Laos; VN, Vietnam; WTH, West Thailand; WKH, West Cambodia. [file 13071_2024_6629_MOESM2_ESM.pdf]

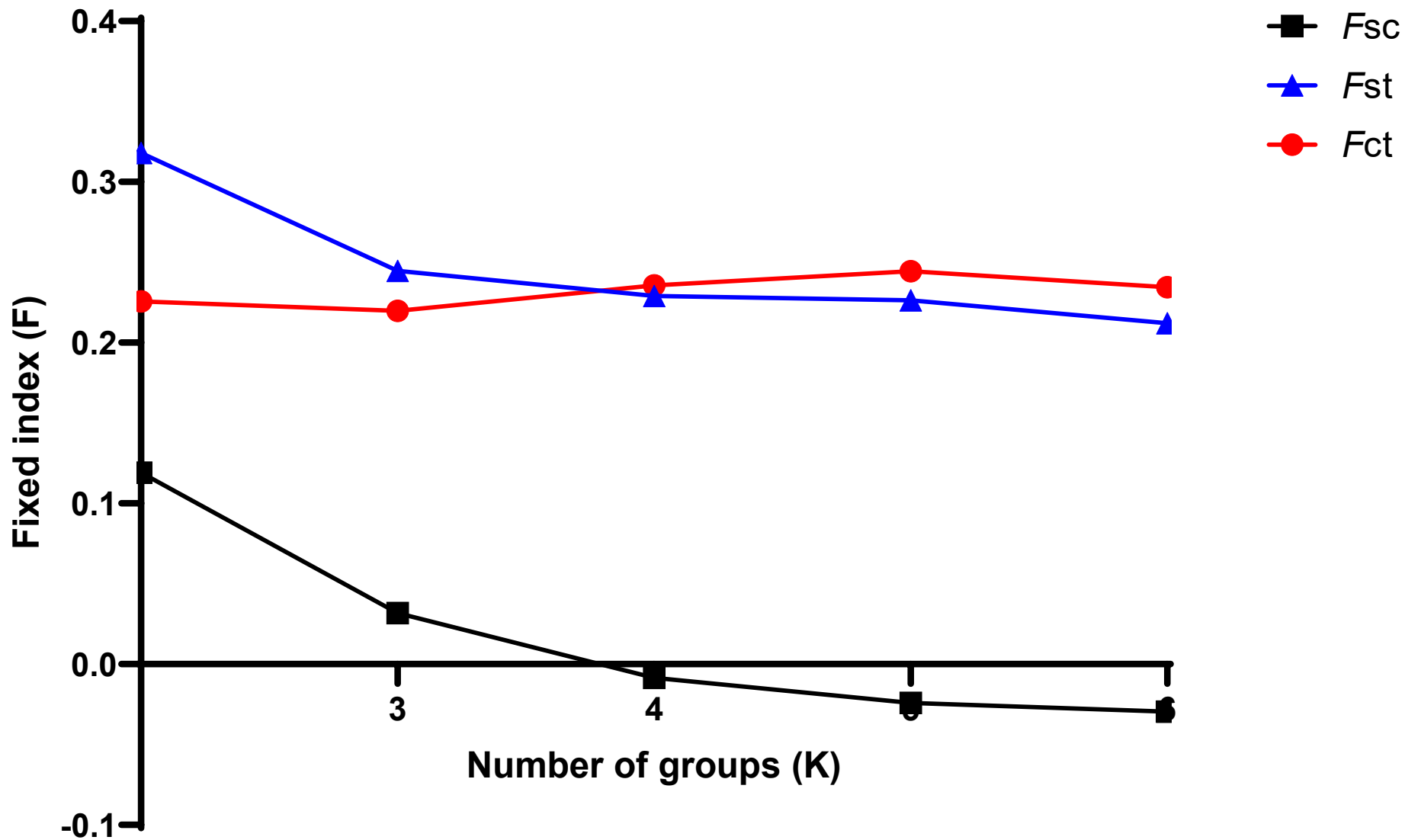

Supplement: Supplementary file 6 — Additional file 6: Figure S2. The Y-axis represents the different F-statistics (FCT, FST, FSC). The red, blue, and black lines represent FCT, FST, and FSC, respectively. The X-axis represents the number of groups (K). SAMOVA estimate was calculated for K = 2-6, with 1000 simulated annealing steps from each of 100 sets of initial starting conditions. [file 13071_2024_6629_MOESM6_ESM.pdf]

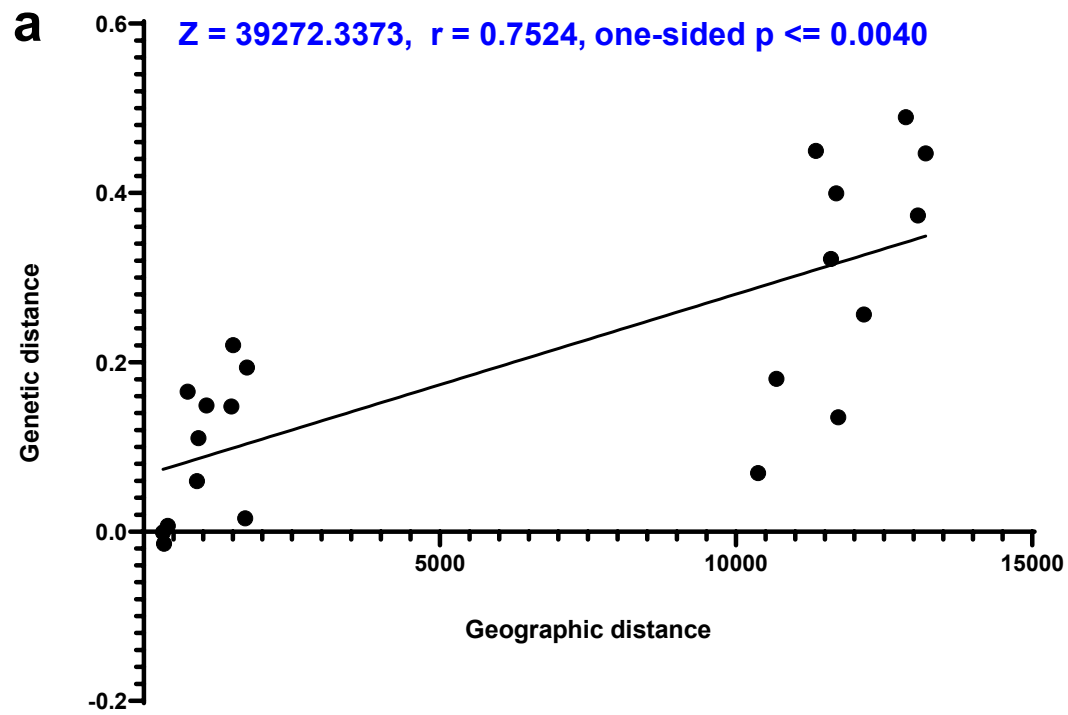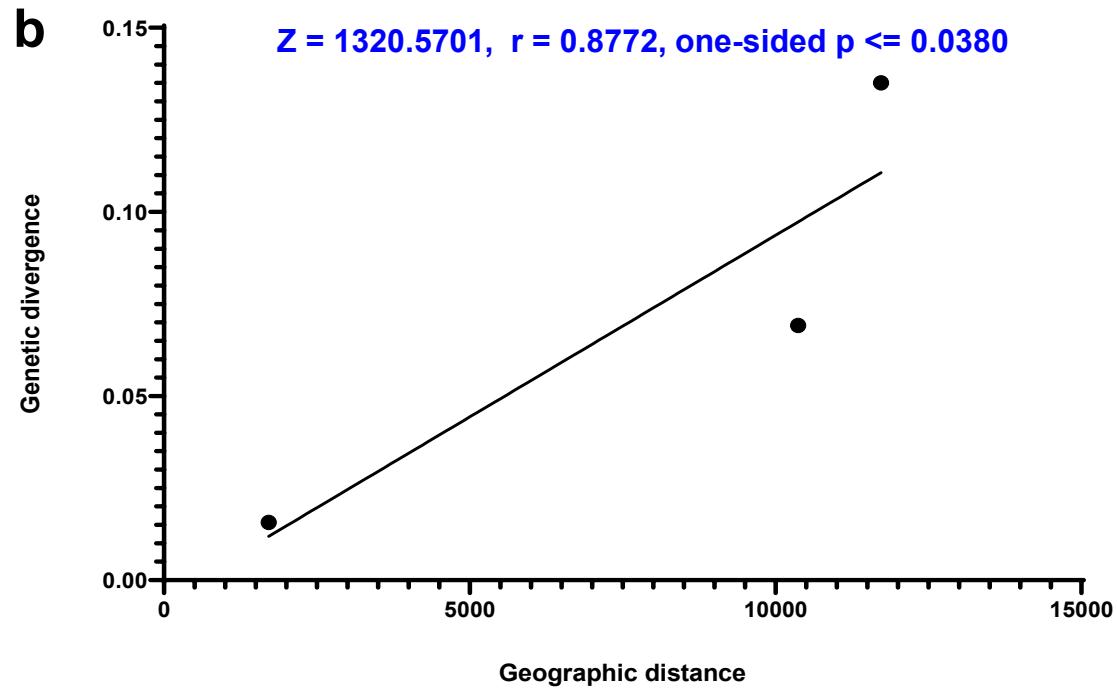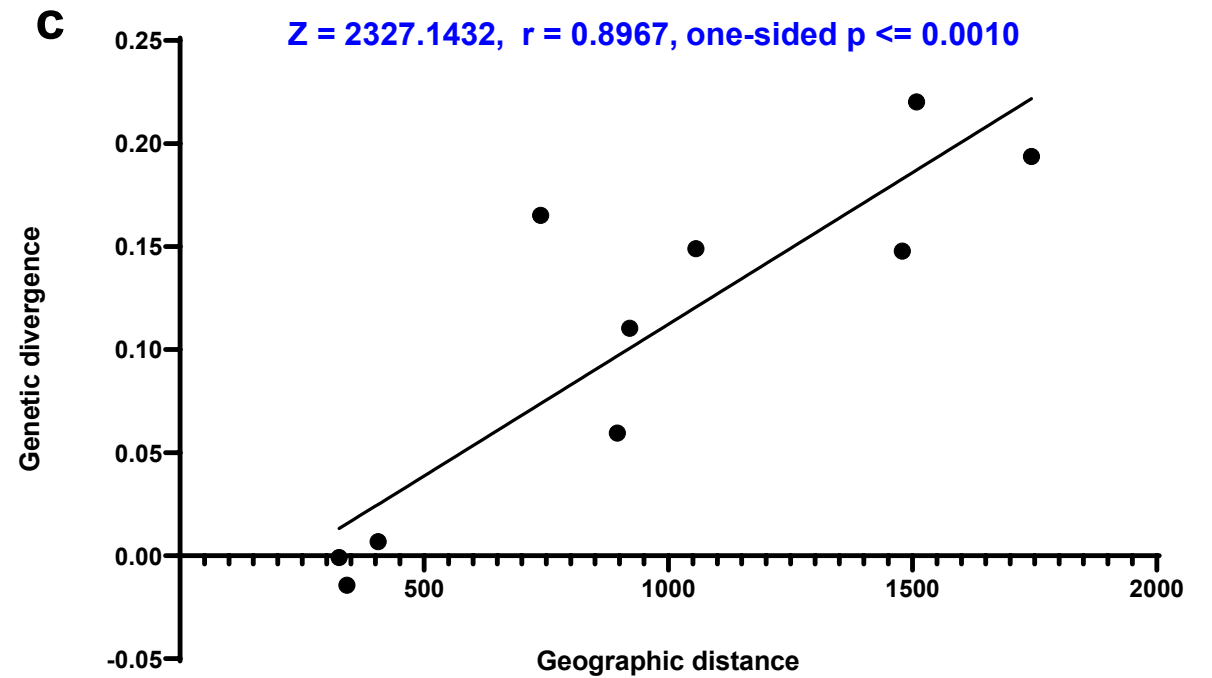

Supplement: Supplementary file 7 — Additional file 7: Figure S3. Isolation by distance, the relationship between geographical and genetic distances based on mt/apico genomes in Plasmodium falciparum populations. Isolation by distance (IBD) was examined using a nonparametric Mantel with the web-based computer program IBDWS v.3.16. (a) IBD of all six populations based on mt/apico genomes; (b) IBD between CMB and WAF populations based on mt/apico genomes; (c) IBD between CMB and SEA populations based on mt/apico genomes. [file 13071_2024_6629_MOESM7_ESM.pdf]

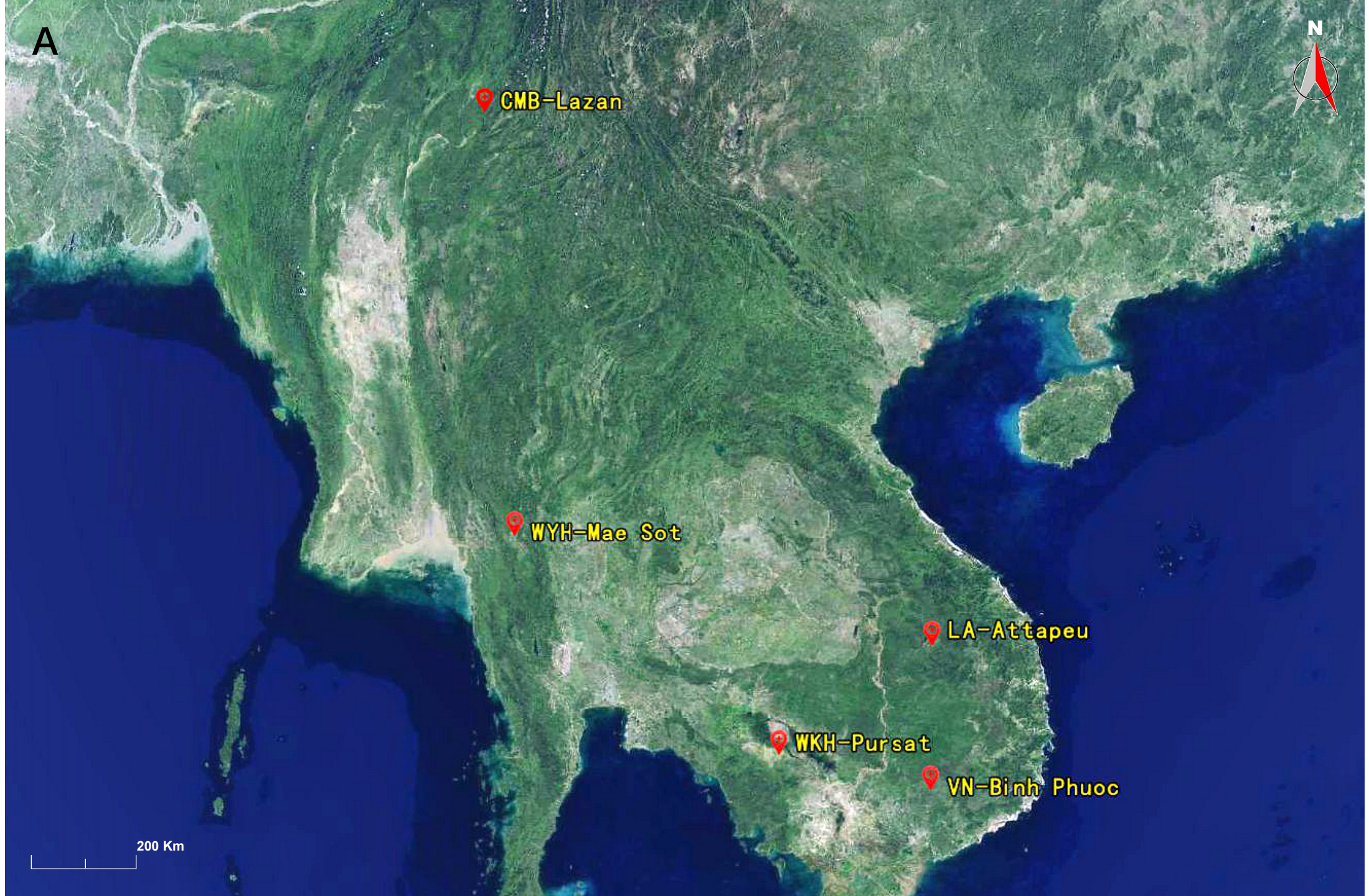

B

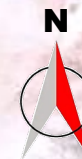

WAF-Banjul, Gambia

WAF-Navrongo, Ghana

300 Km

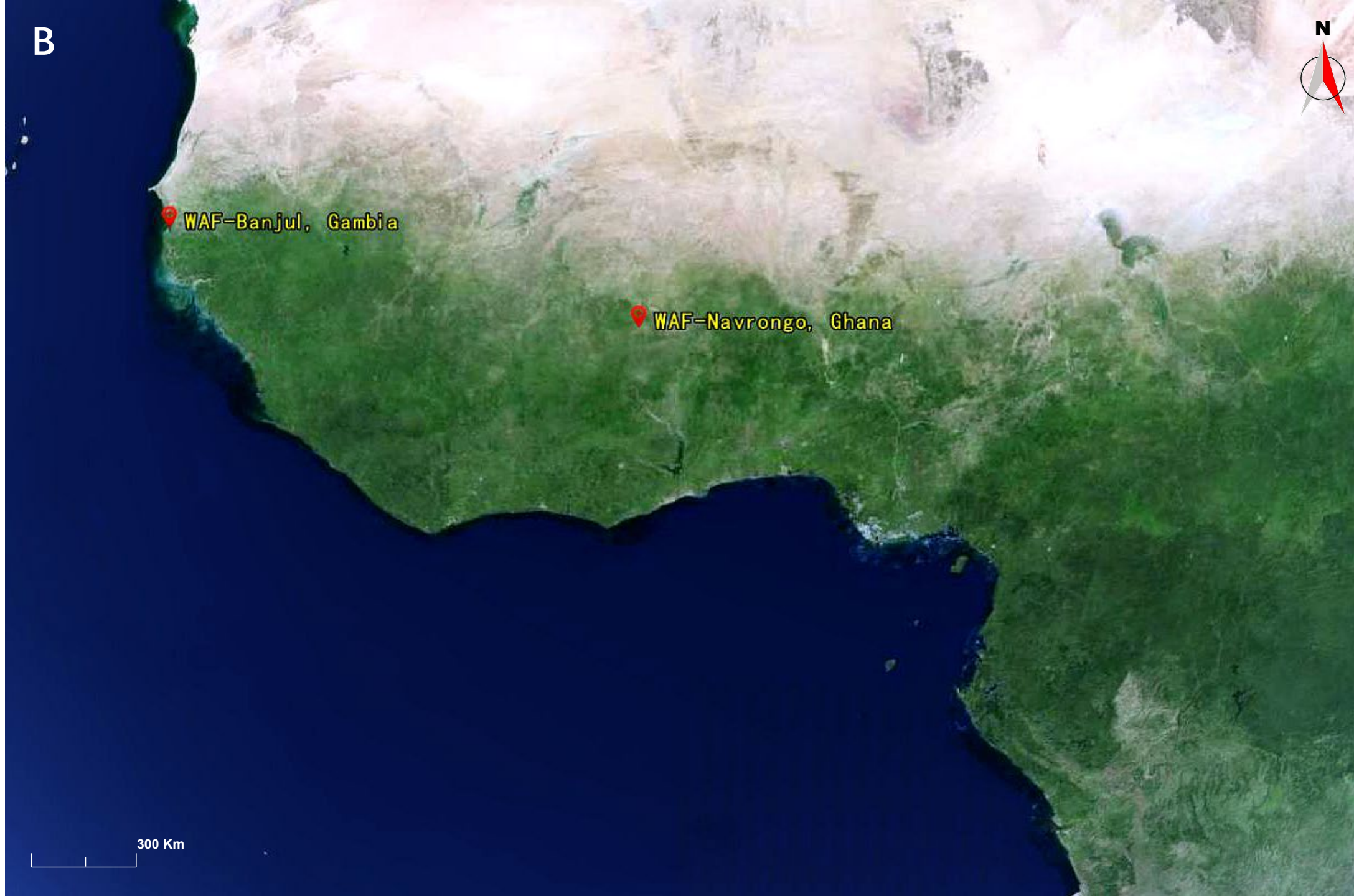

Supplement: Supplementary file 8 — Additional file 8: Figure S4. Map of the populations from different geographical regions. CMB-Lazan, China-Myanmar border; WTH-Mae Sot, West Thailand; LA-Attapeu, Laos; WKH-Pursat, West Cambodia; VN-Binh Phuoc,Vietnam; WAF-Banjul, Gambia; WAFGH-Navrongo, Ghana. The map was prepared using LocaSpace Viewer. [file 13071_2024_6629_MOESM8_ESM.pdf]
