# Supplementary material for: The evolutionary history of Plasmodium falciparum from mitochondrial and apicoplast genomes of China-Myanmar border isolates
Source: Parasit Vectors. 2024 Dec 30;17:548. doi: 10.1186/s13071-024-06629-3 (PMC11686842; doi:10.1186/s13071-024-06629-3)
Supplement: Supplementary file 3 — Additional file 3: Table S2. Analysis of molecular variance (AMOVA) of six Plasmodium falciparum populations based on mt/apico genomes. FCT, fixation index among groups, FSC, among populations within groups, FST, within populations. [file 13071_2024_6629_MOESM3_ESM.docx]

**Table S2. Analysis of molecular variance (AMOVA) of six *Plasmodium falciparum* populations based on *mt/apico* genomes.**

| **Source of variation** | **d. f.** | **Sum of squares** | **Variance components** | **% of variation** | **Fixation index (*P*)** |
| --- | --- | --- | --- | --- | --- |
| Among groups | 2 | 35.396 | 0.27808 Va | 21.99 | *F*_CT_: 0.21987 (*P* < 0 .01) |
| Among populations within groups | 4 | 6.92 | 0.03143 Vb | 2.49 | *F*_SC_: 0.03186 (*P* < 0.05) |
| Within populations | 174 | 166.215 | 0.95526 Vc | 75.53 | *F*_ST_: 0.24472 (*P* < 0.001) |
| Total | 180 | 208.53 | 1.26477 |  |  |
