# Supplementary material for: The evolutionary history of Plasmodium falciparum from mitochondrial and apicoplast genomes of China-Myanmar border isolates
Source: Parasit Vectors. 2024 Dec 30;17:548. doi: 10.1186/s13071-024-06629-3 (PMC11686842; doi:10.1186/s13071-024-06629-3)
Supplement: Supplementary file 4 — Additional file 4: Table S3. Genetic differentiation and gene flow among the geographic groups based on mt/apico genomes. The pairwise FST values and Nm values based on the mt/apico genomes are shown below and above the diagonal, respectively. Characters in bold indicated that the significance was p < 0.05). inf, infinite. CMB, Lazan (China-Myanmar border); WAF-GM, Banjul, Gambia (West Africa); WAF-GH, Navrongo, Ghana (West Africa); WKH, Pursat (West Cambodia); VN, Binh Phuoc (Vietnam); LA, Attapeu (Laos); WTH, Mae Sot (West Thailand). [file 13071_2024_6629_MOESM4_ESM.docx]

**Table S3. Genetic differentiation and Gene flow among the Geographic Groups based on *mt/apico* genomes**

|  | CMB | WAF-GM | WAF-GH | WKH | VN | LA | WTH |
| --- | --- | --- | --- | --- | --- | --- | --- |
| CMB |  | 3.20353 | 6.73109 | 1.77030 | 2.08083 | 2.88320 | 7.89971 |
| WAF-GM | **0.13501** |  | 31.37112 | 0.52139 | 0.61972 | 0.83942 | 1.45083 |
| WAF-GH | **0.06915** | 0.01569 |  | 0.61175 | 0.75191 | 1.05324 | 2.27374 |
| WKH | **0.22023** | **0.48953** | **0.44974** |  | inf | 73.27315 | 2.52711 |
| VN | **0.19374** | **0.44654** | **0.39939** | （0.01437） |  | inf | 2.85510 |
| LA | **0.14779** | **0.37330** | **0.32191** | 0.00678 | （0.00095） |  | 4.02900 |
| WTH | **0.05953** | **0.25630** | **0.18026** | **0.16517** | **0.14903** | **0.11040** |  |
