# Supplementary material for: The evolutionary history of Plasmodium falciparum from mitochondrial and apicoplast genomes of China-Myanmar border isolates
Source: Parasit Vectors. 2024 Dec 30;17:548. doi: 10.1186/s13071-024-06629-3 (PMC11686842; doi:10.1186/s13071-024-06629-3)
Supplement: Supplementary file 5 — Additional file 5: Table S4. Population groups identified by spatial analysis of molecular variance (SAMOVA) algorithm. Significant values *p < 0.05; **p < 0.01; ***p < 0.001, ns: Not significant. CMB, Lazan (China-Myanmar); WKH, Pursat (West Cambodia); VN, Binh Phuoc (Vietnam); LA, Attapeu, (Laos); WTH, Mae Sot (West Thailand); WAF-GM, Banjul (Gambia); WAF-GH, Navrongo (Ghana). [file 13071_2024_6629_MOESM5_ESM.docx]

**Table S4. Population groups identified by spatial analysis of molecular variance (SAMOVA) algorithm**

| **K** | **Population grouping** | ***F*_ST_** | ***F*_SC_** | ***F*_CT_** |
| --- | --- | --- | --- | --- |
| k = 2 | [CMB, WKH, VN, LA, WTH] [WAFGM, WAFGH] | 0.31791^***^ | 0.11916^***^ | 0.22564^ns^ |
| k = 3 | [CMB, WTH] [WAFGM, WAFGH] [WKH, VN, LA] | 0.24472^***^ | 0.03186^*^ | 0.21987^**^ |
| k = 4 | [CMB] [WAFGM, WAFGH] [WKH, VN, LA] [WTH] | 0.22907^***^ | -0.00851^ns^ | 0.23558^**^ |
| k = 5 | [CMB] [WKH, VN, LA] [WAFGM, WTH] [WAFGM] [WAFGH] | 0.22629^***^ | -0.02405^ns^ | 0.24446^*^ |
| k = 6 | [WAFGM] [WAFGH] [WKH, VN] [LA] [WTH] [CMB] | 0.21206^***^ | -0.02944^ns^ | 0.23459^*^ |
